# Dental patients as partners in promoting quality and safety: a qualitative exploratory study

**DOI:** 10.1186/s12903-024-04030-1

**Published:** 2024-04-10

**Authors:** Enihomo Obadan-Udoh, Vyshiali Sundararajan, Gustavo A. Sanchez, Rachel Howard, Siddardha Chandrupatla, Donald Worley

**Affiliations:** 1 Department of Preventive and Restorative Dental Sciences, University of California San Francisco, School of Dentistry, 707 Parnassus Avenue, D3214, Box #1361, San Francisco, CA 94143 USA; 2 Ibn Sina Community Dental Clinic, Houston, TX USA; 3 HealthPartners Institute, Bloomington, MN USA

**Keywords:** Dental care, Healthcare quality, Patient safety, Qualitative study, Patient engagement

## Abstract

**Objective:**

Active patient involvement in promoting quality and safety is a priority for healthcare. We investigated how dental patients perceive their role as partners in promoting quality and safety across various dental care settings.

**Methods:**

Focus group sessions were conducted at three dental practice settings: an academic dental center, a community dental clinic, and a large group private practice, from October 2018-July 2019. Patients were recruited through flyers or word-of-mouth invitations. Each session lasted 2.5 h and patients completed a demographic and informational survey at the beginning. Audio recordings were transcribed, and a hybrid thematic analysis was performed by two independent reviewers using Dedoose.

**Results:**

Forty-seven participants took part in eight focus group sessions; 70.2% were females and 38.3% were aged 45-64 years. Results were organized into three major themes: patients’ overall perception of dental quality and safety; patients’ reaction to an adverse dental event; and patients’ role in promoting quality and safety. Dental patients were willing to participate in promoting quality and safety by careful provider selection, shared decision-making, self-advocacy, and providing post-treatment provider evaluations. Their reactions towards adverse dental events varied based on the type of dental practice setting. Some factors that influenced a patient’s overall perception of dental quality and safety included provider credentials, communication skills, cleanliness, and durability of dental treatment.

**Conclusion:**

The type of dental practice setting affected patients’ desire to work as partners in promoting dental quality and safety. Although patients acknowledged having an important role to play in their care, their willingness to participate depended on their relationship with their provider and their perception of provider receptivity to patient feedback.

**Supplementary Information:**

The online version contains supplementary material available at 10.1186/s12903-024-04030-1.

## Introduction

In recent years, there has been a surge in research related to dental quality and safety from various parts of the world [1–11]. These studies have investigated how adverse events occur in dentistry, identified methodologies for detecting adverse safety events, and focused on developing strategies to reduce the occurrence of adverse events [3, 5, 6, 10–14].

Patient involvement in quality and safety-promoting activities is an emerging area of interest. Dentistry findings from several studies suggest that patient reports can provide meaningful insight and breadth regarding the quest to understand such adverse events [15–19]. In dentistry, previous studies have demonstrated that patients are apprehensive about safety at the dental office and are willing to participate in activities that promote quality and safety when properly engaged by providers [7, 8, 15–17]. However, majority of these studies focused solely on patients attending a single academic institution. We proposed a study that recruited patients from three different dental care settings to provide an array of diverse perspectives. Through this study, we assessed how dental patients perceive their role as partners in promoting quality and safety across various dental care settings.

## Methods

### Study sites

The study was conducted at three different dental practice settings: an academic dental center (Site U), a community dental clinic (Site I), and a large group dental private practice (Site H). Site H is in Minnesota, while both Sites U and I are located in Texas. The academic dental center (Site U) consists of a pre-doctoral teaching clinic, a resident/postgraduate clinic, and a faculty group practice. Patients visiting this center are from diverse racial and ethnic backgrounds. The community dental clinic (Site I) is comprised of a network of small to medium-sized dental offices scattered throughout the city. Affiliated with a religious non-profit organization, this site primarily serves low income and indigent families who lack access to health care. The large group dental private practice (Site H) encompasses over 70 dentists working across 21 dental office locations. The group serves a large range of patients from various demographic and socioeconomic backgrounds.

### Study participants

Participants were recruited using a purposive sampling approach. Patients from the study sites were invited to participate through flyers and word-of-mouth invitations. Interested participants were screened by a local site coordinator to confirm that they met the following inclusion criteria: English-speaking, over 18 years of age, able to give informed consent, and attended at least one dental visit at a participating site. Enrolled participants selected one of eight focus group sessions: three of which were hosted at the academic dental center, three at the large group dental practice, and two at the community dental clinic. Ethical approval was obtained from the UCSF institutional review board (#18–25467). A unique participant’s ID was created for each participant using their site name and sex. Females were represented by “X” and males by “Y”.

### Study procedures

Eight focus group sessions were held from October 2018 through July 2019. Researchers were provided with a quiet conference room within each dental site to host the sessions. Sessions lasted about 2.5 h and were recorded using two voice recorders. Participants completed an anonymous demographic questionnaire, an informational survey, and an evaluation form at the beginning or end of the session. Prior to the start of each session, ground rules were provided to participants about taking turns, refrainment from disclosure of comments to external parties, and the need to tolerate dissenting opinions. Name introductions were also done prior to starting the recording to ensure privacy. Participants provided verbal consent after reading the study information sheet. All audio recordings were stored safely on a password-protected laptop and de-identified for analysis. Participants received a $30 gift card along with food and beverage in appreciation of their time. The first author (EO-U) served as the lead facilitator/moderator for all the sessions. There were no conflicts of interest, given the authors do not provide patient care at any of the participating dental institutions.

### Study instrument

A focus group discussion guide and informational survey were developed using questions from our previous qualitative study and publications by Davis et al. [8, 16, 17]. The discussion guide consisted of eight main topics with sub-topics and probing questions (see Additional file 1). The topics were:

1. Patients’ understanding about “patient safety” and “quality”
2. Current practices to ensure high quality care in dental care settings
3. Perceptions about dental patients contributing to dental quality and safety
4. Approaches to improve patient engagement in dental quality and safety activities
5. Factors affecting patients’ willingness to participate in dental quality and safety activities
6. Factors affecting the reporting of poor quality or adverse safety incidents
7. Features of a “safe” incident reporting system or platform
8. Patient considerations about the quality and safety of dental offices

The 45-item informational survey comprised six sections that assessed interactional and non-interactional behavior between patients and the dental care team including: asking factual or challenging questions, notifying the team, providing information, gaining information, and adverse event reporting.

### Data analysis

Frequencies and descriptive statistics for demographic characteristics of the participants and survey responses were calculated, and audio recordings from the focus group discussions were professionally transcribed using Rev.com. The transcripts were verified against the original audio recordings for completeness by one co-author (VS).

Using Dedoose, two co-authors (VS, GS) independently analyzed each transcript using the following steps:

- *Repeated reading*: After verifying the transcripts, data was read multiple times and memo writing was performed in Microsoft® Word. The research question served as a guide during this step.
- *Data coding*: Bucket codes were created, and relevant texts from transcripts were selected for initial codes and coded for as many potential themes as feasible.
- *Exploring for themes*: After completion of the initial coding process, a list of different codes identified across the data was generated. Within a code, common themes based on similarities, differences, topics, demographics, and approaches were extracted from the content of the focus group discussions. Opinions expressed by individuals that diverged from group consensus were also identified.
- *Discussing themes and sub-themes*: Both primary coders discussed their initial codes. Similar or duplicate codes were merged into sub-themes. All codes were organized into three major themes based on the study objectives:
  - Patients’ overall perception of dental quality and safety (Theme 1)
  - Patients’ reaction to poor quality dental care and adverse events (Theme 2)
  - Patients’ perception of their role in promoting quality and safety (Theme 3)
- *Tie-breaking*: Any code names or code applications that were discordant between both reviewers, were discussed with a third researcher (EO-U) who acted as a tiebreaker in finalizing the themes, sub-themes, and codes. This was only needed once in the study.

## Results

### Demographic characteristics

A total of 47 patients (*n* = 20 (Site I), *n* = 16 (Site H), *n* = 11 (Site U)) were successfully recruited to participate in eight focus group sessions (Table 1). Participants in the focus group sessions were mostly female (70.2%), and Caucasian (40.4%). Asians (23.4%) and Latinos (21.3%) were the second and third largest ethnicities represented, respectively. The educational level varied among participants, with nearly 50% having at least a bachelor’s degree. Most participants were aged between 25 – 64 years (68.1%).

**Table 1:** Demographic characteristics of focus group participants

	**Site 1 (I) (N, %)**	**Site 2 (H) (N, %)**	**Site 3 (U) (N, %)**	**Total Number (N)**	**Total Percentage (%)**
**Sex**					
Male	7 (35)	3 (18.8)	4 (36.4)	14	29.8
Female	13 (65)	13 (81.2)	7 (63.6)	33	70.2
**Age groups (in years)**					
18–24	4 (20)	-	-	4	8.5
25–44	9 (45)	3 (18.8)	2 (18.2)	14	29.8
45– 64	5 (25)	10 (62.4)	3 (27.3)	18	38.3
65 +	-	3 (18.8)	6 (54.5)	9	19.1
Prefer not to answer	2 (10)	-	-	2	4.3
**Race**					
White	3 (15)	12 (75)	4 (36.3)	19	40.4
Black/African American	-	2 (12.4)	4 (36.3)	6	12.8
Asian	11 (55)	-	-	11	23.4
Hispanic/ Latino	6 (30)	1 (6.3)	3 (27.2)	10	21.3
No answer	-	1 (6.3)	-	1	2.1
American Indian/ Alaska Native/ Native Hawaiian/ Other Pacific Islander	-	-	-	-	-
**Education**					
< High School	6 (30)	-	1 (9.1)	7	14.9
High school diploma or technical degree	6 (30)	3 (18.8)	5 (45.4)	14	29.8
Associate/Bachelor’s degree	4 (20)	9 (56.2)	4 (36.4)	17	36.2
Master’s/ Professional/Doctoral degree	4 (20)	4 (25)	1 (9.1)	9	19.1
**Dental visit in last year**					
Yes	10 (50)	15 (93.7)	10 (90.9)	35	74.5
No	10 (50)	1 (6.3)	1 (9.1)	12	25.5
**Total**	20	16	11	47	100

### Informational survey

The informational survey results were analyzed using descriptive statistics (Table 2). Patients were asked to assess willingness to engage in various activities at the dental office using a Likert scale (1 = “Definitely Will” through 7 = “Definitely Not”). Thus, lower mean scores represent patients’ willingness to play an active role in promoting quality and safety and higher scores represented an unwillingness to engage in that activity. While patients appeared willing and comfortable to ask factual questions from dental assistants/hygienists and dentists, patients were less willing to ask challenging questions of such providers unless the provider encouraged it (Table 2; Sections 1a, 1b, 2a, and 2b). Patients also reported feeling relatively willing to notify their providers as concerns arose regarding their care (Table 2; Sections 3a, 3b). Furthermore, while patients were willing to provide or gain pertinent information about their care or dental office, they were less willing to participate in reporting an adverse event occurrence unless it was encouraged by the dental care team (Table 2; Section 4, 5, and 6). We defined an adverse event as “the occurrence of any event that the patient perceived as negative or harmful while receiving care”. There was no significant difference between patients’ willingness to engage in safety behaviors with different members of the dental care team (i.e. dental assistant/ hygienist versus dentist).

**Table 2:** Descriptive statistics for informational survey

Item description	Mean	SD
**1a. Factual Questions to a Dental Assistant/Hygienist (Interactional Behavior)**		
Would you ask a dental assistant/hygienist: How long will this dental procedure last?	2.06	1.38
Would you ask a dental assistant/hygienist: How long will the pain last after the procedure?	2.11	1.52
Would you ask a dental assistant/hygienist: What signs should I look out for if my teeth/gums are not healing as they should?	2.23	1.52
Would you ask a dental assistant/hygienist: When can I resume eating and drinking?	1.83	1.5
Would you ask a dental assistant/hygienist: How is the procedure (e.g. scaling and polishing) performed?	2.91	1.54
If a dental assistant/hygienist encouraged you to ask the above questions (e.g. by saying “it’s ok to ask staff questions”), would you be more willing to ask these questions?	1.98	1.34
**1b. Factual Questions to a Dentist (Interactional Behavior)**		
Would you ask a dentist: How long will the pain last after the procedure?	1.82	1.34
Would you ask a dentist: How long will this dental procedure last?	2.13	1.28
Would you ask a dentist: When can I resume eating and drinking?	1.82	1.27
Would you ask a dentist: How is the procedure (e.g. root canal treatment) performed?	2.4	1.63
Would you ask a dentist: What signs should I look out for if my teeth/gums are not healing as they should?	2.07	1.44
If a dentist encouraged you to ask the above questions (e.g. by saying “it’s ok to ask dentists’ questions”), would you be more willing to ask these questions?	1.87	1.28
**2a. Challenging Questions to a Dental Assistant/Hygienist (Interactional Behavior)**		
Would you ask a dental assistant/hygienist: Can you check that you have the correct tooth site/location for my procedure?	3.26	1.81
Would you ask a dental assistant/hygienist: How many of these procedures have you performed?	4.09	1.85
Would you ask a dental assistant/hygienist: Why are you using that instrument/piece of equipment?	4.49	1.5
Would you ask a dental assistant/hygienist: Have you washed your hands?	4.75	1.72
Would you ask a dental assistant/hygienist: What are the risks, benefits, and alternatives of this procedure?	2.75	1.63
If a dental assistant/hygienist encouraged you to ask the above questions (e.g. by saying “it’s ok to ask staff questions”) would you be more willing to ask these questions?	2.34	1.48
**2b. Challenging Questions to a Dentist (Interactional Behavior)**		
Would you ask a dentist: Why are you using that instrument/piece of equipment?	4.15	1.7
Would you ask a dentist: Can you check that you have the correct tooth site/location for my procedure?	4.13	1.6
Would you ask a dentist: How many of these procedures have you performed?	4.47	1.4
Would you ask a dentist: Have you washed your hands?	4.6	1.78
Would you ask a dentist: What are the risks, benefits, and alternatives of this procedure?	2.31	1.31
If a dentist encouraged you to ask the above questions (e.g. by saying “it’s ok to ask dentists’ questions”) would you be more willing to ask these questions?	2	1.1
**3a. Notifying Dental Assistant/Hygienist (Interactional Behavior)**		
Would you notify a dental assistant/hygienist if you thought your gums/mouth had become infected after a procedure?	1.71	1.4
Would you notify a dental assistant/hygienist if they had the dental record/radiograph of the wrong patient pulled up while discussing your treatment plan?	1.59	1.24
Would you notify a dental assistant/hygienist if you did not receive the results of a biopsy test for an oral swelling/mass?	1.53	0.97
Would you notify a dental assistant/hygienist if you thought an error had occurred in your care?	1.72	1.17
Would you notify your dental assistant/hygienist if there has been a significant change in your medical history?	1.57	0.85
For the above problems and concerns, if a dental assistant/hygienist said to you “it’s ok to notify me of any of these problems or errors”, would you be more willing to do this?	1.68	0.96
**3b. Notifying Dentist (Interactional Behavior)**		
Would you notify a dentist if you did not receive the results of a biopsy test for an oral swelling/mass?	1.34	0.71
Would you notify a dentist if you thought an error had occurred in your care?	1.5	1.06
Would you notify a dentist if you thought your gums/mouth had become infected after a procedure?	1.31	0.78
Would you notify a dentist if they had the dental record/radiograph of the wrong patient pulled up while discussing your treatment plan?	1.4	0.93
Would you notify your dentist if there has been a significant change in your medical history?	1.39	0.69
For the above problems and concerns, if a dentist said to you “it’s ok to notify me of any of these problems or errors”, would you be more willing to do this?	1.75	1.01
**4. Information Provision (Non-Interactional Behavior)**		
Would you be willing to bring into the dental office, medications that you are taking and a list of allergies?	1.96	1.01
If a dentist encouraged you to bring into the dental office, medications and a list of allergies, would you be more willing to do this?	1.62	1.6
If a dental assistant/hygienist encouraged you to bring into dental office, medications and a list of allergies, would you be more willing to do this?	1.59	1.4
**5. Information Gain (Non-Interactional Behavior)**		
Would you want to be given information to help you decide which dental office had the highest safety record for your treatment?	1.7	1.03
If a dentist encouraged you to look at information to help you decide which dental office had the highest safety record, would you be more willing to do this?	1.52	0.86
If a dental assistant/hygienist encouraged you to look at information to help you decide which dental office had the highest safety record, would you be more willing to do this?	1.64	0.88
**6. Reporting (Non-Interactional Behavior)**		
If you experienced an error in your care, would you report this to a national reporting system?	3.02	1.55
If a dentist encouraged you to report an error you experienced in your care to a national reporting system, would you be more willing to do this?	2.09	1.34
If a dental assistant/hygienist encouraged you to report an error you experienced in your care to a national reporting system, would you be more willing to do this?	2.04	1.17

### Theme 1: patients’ overall perception of dental quality and safety (Table 3, Section 1)

**Table 3:** Illustrative quotes of patient experiences

Theme	Sub-theme	Illustrative quote
**Section 1.** Patients’ Overall Perception of Dental Quality and Safety	Provider Training and Qualifications	***I14X:*** *“For me, the doctors, make sure they have the right degree or masters, or they went through all the rules and studies that need to [be] completed before they start practicing.”* ***H1X:*** *“What are their education? What's your background? What schools did you go to? And did you go to what school? There's a difference between going to the University of [State], then someone who went to a two-year college. You know what I'm saying?”* ***H15X:*** *“I always feel comfortable when the provider tells me how long they've been doing this or sometimes I will ask them or just kind of gauging by your age, like whether […] they like their job? Do they enjoy what they're doing?”* ***H1X:*** *“Well, first of all, I want to know if they've been, of course, in business a long time. If they haven't been in business long, if they've been in business for a year, I would probably not go to that provider… And so other things that will be a factor for me is do you have a lot of patients that have come to you and [do] they like you? What is your rating?”* ***U5X:*** *“Maybe my other concern is the fact that we have this pre-assumption in the back of our minds that we are coming to a dental school, they are students. So, already, in your mind, you have already kind of put like a question, that this is the quality of care you are going to receive. You're giving them the benefit of doubt that "Okay, they are students in as much as there's a professor helping them or somebody behind them or shadowing them," but you are looking at the fact that they're not yet there. So, you have already adjusted your level of expectation…”* ***U1X:*** *“Actually, I think I felt safer. I didn't know it beforehand coming to an academic dental office … Because you have, like, a double-check. You have the student who's been groomed on what's supposed to happen, and then you also have a teacher that's overlooking it. So, there's like, two layers of safety there, and when you go to a dental office, a local or a private one, I don't know that there's anybody that really oversees, say, the hygienist or the dentist. Nobody oversees the dentist.”*
	Communication	***U2Y:*** *“I guess by the information they give you. I mean, when they first meet you, the information they give you is the way I would think they can assure you that they're going to be safe in what they do. Like, at the dentist school, the students explained, the one I had explained to me exactly what she was going to do before she did it, and it kind of made me feel okay.”* ***I15X*** *: “What for me is mostly concern[ing] when I go to the dentist, [is] that [the] dentist communication should be very honest. Because today I did my root canal … and I did my x-ray before. Before that, I went to three or four places to do my x-ray, different dentists w[ere telling me different things. It was three fillings with steel, later four. If we can restore the tooth they say, "No, you have only extractions options."*
	Cleanliness and Clinic Environment	***U1X:*** * “Well, I think maybe by observation, you would want to feel like their instruments were clean. Now how you would go about knowing that I'm not sure. You assume it, I think. And you assume that their hands… Lot of times they wear gloves.”*
	Durability of Dental Treatment	***U12X:*** * “…Nothing's made like it used to be done in the old days…, sometimes if you had a cap put on, most of the time it would last for a long, long time. And now days it seems like it could be only good for three years or so; or four years and I have to redo that cap.”*
**Section 2.** Patients’ Reaction to Poor Quality Dental Care and Adverse Events	Breach of Trust	***Researcher:*** *“Would you still get care from that provider?”* *U5X:* *“Oh, no”* *U3X:* *“I'll think about it”* *U4Y:* *“I wouldn't… I would stop procedure. If you don't know whether you're supposed to [treat] the right side and you started on the left side, I'd thank you, but I wouldn't… And then, I would say, No. I'm sorry. I'm going to go somewhere else."* *I17X: “I just wanted to say like a year ago I went to a dentist's office, and I sat in that chair for 30 min and she must have given me 30 to 50 x-rays because she couldn't get it right and I almost literally walked out because you've got that thing in your mouth, and I hate that thing anyway. I literally almost got up and said, never mind. I don't want this anymore. But I definitely would never go back.”* *I7X:” I went through a very bad experience during my extraction so right now I have this concern, I'm going to ask them if they are like qualified are they students or what? I never asked them…Yeah. I have that fear now. I'm very scared now, I have this implant pending. I'm not doing it just because of that fear.”*
	Fear and Embarrassment	***I7X*** *: “… it happened in the evening till the next afternoon… because I had that fear here and the pain here and there…they put the bone graft and of course I had stitches. Everything aggravated, I was scared to till date… I didn't know till last year and I'm not doing going for implant. I'll just leave [it] like that.”* ***U11X:*** *“…but sometimes you don't really understand, this is what's going to happen…you don't even know what some of these words mean, but like I say, you're afraid to speak up because you're like, "Am I going to look stupid?" I think that little confidence is like, "Well they know what they're doing. I don't know what I'm talking about so I don't want to look like I'm less of a person because I don't know how to communicate my needs.”*
	Clinic Response to Adverse Events	***U6X:*** *“… the attitude also matters because if they are understanding and they are trying to work with you, you know error is human. So, you try to work with them. But if they are adamant about what they have done and they don't want to work with you, then yeah, you seek legal advice and take it from there.”* ***U2Y:*** *“Well, like she said. Everybody can make a mistake. But it depends on the attitude about the mistake. Like, if they have the right attitude, "I'm sorry. I'll take care of it," that's okay. But if they're almost like, "Hey. I don't have nothing to do with that part," then you move to another… your attitude's going to change. It's all about attitude.”* ***H6Y:*** *“I don't know if there is like a patient advocate. Who would you call or ask]?”* ***I16X*** *: “I would say keep phone numbers open because of course the greatest weapon is what way of communication? Try to have somebody in the office who can take that feedback, not just the doctor itself. It you don't feel comfortable talking to your doctor, then get somebody that can… get somebody that is a feedback person. have them there.”* ***H1X:*** *“Because if I was experiencing an issue with a doctor and I did not feel I was getting the right care or I wasn't able to ask the right questions, I would be able to advocate, get my advocate involved so that he will understand what I need, what my needs are before I walk through the door. And so that way I can get my needs met.”*
**Section 3.** Patients’ Perception of Their Role in Promoting Quality and Safety	Rationale for Participation	***U11X:*** *"… If this is happening, this could happen to me or somebody else."; “So, I personally …make it a big deal when I see something that is out of the normal or I'm concerned about, even if it's not personally dealing with me, I tend to report it. Because I feel like if this is happening, who knows what else is happening and who it's happening to?”* ***H1X:*** *“… I'm an advocate for myself, not everybody can do that. I advocate for myself because I need to know that I'm getting the best care and if I feel like I need a second opinion, I'm going to do that too… I even had this situation where I went to a different provider because I felt like I wasn't getting my needs met, and I did go and I got my needs met. It's all about being able to say what you need, not everybody can do that, I can.”*
	Timing of Participation	***U9X:*** *“Well, yeah. If something happens, something like that day with my root canal. Yeah. That was not good. And yeah, I let him know in a hurry. This is not going to work. Cause I went back and said, okay, I paid you up front, and you didn't do the job. I want at least half of my money back.”; “Patients should do it when they're all done with whatever you're having done… [not] asking their opinions later….”* ***I11X:*** *“I would prefer not to have it right away, but maybe wait until the next day to ensure the patient is able to provide accurate feedback.”*
	Format of Participation	***U6X:*** *“Sometimes when you visit a physician, they have these surveys at the end of the visit. It's just a small note, a slip that they tell you to give your comment……. maybe it's not going to be like the whole year but periodically, you can put those boxes that people can write comments on how their visit was, so they can put it down.”* ***H14X:*** *“Yeah. Possibly or that little reminder card. Why we want to continue improve that quality. If you have any concerns, please let us know. We want to learn from you…and encouraging people with concerns that they really want to hear them…”* ***I15X:*** *“If the patient really cared about their health, they care about their self, they will. I was so desperate to let them know what I was thinking so I wrote a letter. So, I think if patients want to, they will.”*

#### Provider training and qualifications

Patients trusted that providers who possessed the proper dental licenses and credentials had received adequate training and education needed to provide high quality dental care. Patients found reassurance in public displays of the provider certificates and necessary clinic approvals, many of whom also inquired the names of their provider’s dental school and their experience performing certain procedures.

Patients at the private dental practice and community health center placed more value on the providers’ experience, years of practice, and reviews through websites, such as Yelp. These patients preferred providers who were not recent graduates; however, they also wanted providers to be familiar with recent clinical procedures and guidelines. In contrast, patients who received care at the academic center had tempered expectations about quality. These patients were comfortable with students’ performing treatments because supervising faculty oversaw every procedure.

#### Communication

Patients emphasized the importance of clear communication in promoting trust between patients and their dental providers. Participants also wanted providers to be honest about the necessary procedures, and their comfort level with performing those procedures. They preferred that providers educated them appropriately about their oral health and the necessary dental treatment using various methods (e.g., pictures, pre-visit videos, after-visit summaries) to facilitate informed decision making.

Unsurprisingly, exemplifying polite and courteous behavior (good chairside manners) as well as establishing a good rapport were often associated with high quality dental care. Older patients preferred more direct or in-person communication, while younger patients preferred more on-demand or virtual communication. Although the preferred communication methods varied, the theme of clear communication was unanimously expressed as a marker of quality dental care across all dental practice settings.

#### Cleanliness and clinic environment

Irrespective of the dental practice setting, most patients believed that cleanliness and sanitation were important indicators of dental quality and safety. Markers of cleanliness included: sterile instruments within sealed pouches, clean restrooms, clean floors and office space, constant use of gloves around patients, and the physical appearance of dental staff. Markers of uncleanliness included: foul smells, blood on syringes or instruments used for previous patients left lying around, opened sterilization pouches, and re-using dropped instruments.

#### Durability of dental treatment

Given patients expected their procedures to last, there was a perception of poor-quality care if patients needed to return for repeat procedures or treatment within a short time period.

#### Summary

Dental patients based their overall perception of dental quality and safety on the professional credentialing of providers, their individual dental care experience, the quality of provider-patient communication, the cleanliness of the dental office environment, and the durability of their treatments. Although patients from all dental settings emphasized the importance of cleanliness, perception of other sub-themes varied by dental practice setting. Whereas patients attending the academic dental center gave the benefit of doubt to the student providers and assumed a degree of risk with receiving poor quality dental care and experiencing adverse events, patients at the private dental office placed more emphasis on the quality of provider training and qualifications.

### Theme 2: patients’ reaction to poor quality dental care and adverse events (Table 3, Section 2)

#### Breach of trust

When a patient visits a healthcare provider, they expect to receive adequate treatment to ensure good health. In the focus group sessions, patients expressed that experiencing an adverse event negatively impacted their ability to trust their providers, leaving them uncertain about how best to proceed. Some patients chose not to return to the culpable provider and looked for service elsewhere. Others decided to stop seeking dental treatment altogether due to the anxiety from the negative experience.

#### Fear and embarrassment

During the focus group sessions, some patients indicated that they were afraid to speak up after a perceived adverse event (i.e., received poor quality care or were harmed by dental treatment). Patients also reported feeling embarrassed for their lack of dental health literacy regarding the dental procedure when an incident occurred.

#### Clinic response to adverse events

Good communication and provider attitude influenced how patients reacted to adverse situations. Patients expressed the importance of dental practices providing a patient support advocate with whom patients can voice their concerns and/or opinions. However, some patients were concerned that speaking with a patient advocate could lead to provider backlash.

#### Summary

Patients had varying reactions to adverse events and receiving poor quality dental care. Most patients across all institutions expressed that they received adequate support from the provider/clinic team whereas others (predominantly from the community health center) described a reluctance to report their experience due to fear of retribution.

### Theme 3: patients’ perception of their role in promoting quality and safety (Table 3, Section 3)

#### Rationale for participation

Dental patients had mixed reactions about their role in promoting dental quality and safety. Those who were positively disposed towards engagement activities believed that participation made them feel empowered about their care, stay informed about their choices, and play an active role in improving their oral health. Some believed reporting safety incidents helped make dental care safer for everyone. Patients who were hesitant about their role in promoting dental safety indicated they felt active participation was unnecessary unless they had received poor quality or unsafe dental care.

#### Timing of participation

Patient willingness to participate in different types of engagement activities depended on the dental care setting. For example, patients at the academic dental center preferred to deliver feedback immediately or shortly after receiving dental treatment, whereas patients at the private dental practice and community-based dental clinic preferred to wait until after they left the clinic or completed their procedure.

#### Format of participation

Quality and safety-promoting activities that patients were willing to participate in included: advocating for self when they felt that something was going awry, actively tracking their medical/dental health information, writing reviews, participating in dental research, educating themselves about their oral health conditions and dental procedures, and asking their dental providers questions about their dental procedures or safety practices (e.g., hand washing, sterilized instruments, post-procedural instructions). Patients were more hesitant to ask questions that could potentially appear confrontational. Conversely, patients indicated they were more comfortable speaking when providers invited and encouraged their feedback.

Patients also expressed willingness to participate in focus groups, which provide opportunities to voice opinions without fear of any repercussions from the dentist. Others preferred to have fill out surveys, provide comment cards, or speak directly with a dentist or office staff. Patients from the private practice setting appeared more willing to be involved in promoting the quality and safety of their experiences.

Patients preferred different types of communication based on their demographic information. Older patients with less experience using computers preferred printed materials. No comparable differences were observed between male and female participants in the focus groups; although males participated less often than females in the study.

#### Summary

Most patients, especially those at the academic institution, were willing to participate in activities that promote better dental quality and safety and offered various strategies for increasing patient engagement. However, a few patients from the private group practice expressed concerns about “over-engagement” and suggested that patients should be left alone unless they experienced an adverse event. Reactions varied by dental practice setting, with patients at the private dental office indicating that they received more support when things went wrong than patients at the community dental clinic or academic dental center.

## Discussion

This study investigates factors that influence dental patients’ perceptions of their role in promoting quality and safety across various dental care settings. We used multiple focus group sessions to define patients’ understanding of quality and safety and summarize their past experiences of receiving poor quality or unsafe dental care. Given the scarcity of literature on this topic, this study provides novel insight from three diverse dental care settings to jumpstart the conversation about quality of care from the patient’s perspective.

Although patient safety is a complex, multifactorial matter [1, 18], our study and others have found that patients consider cleanliness a key component of patient safety. Congruent with our previous work [8], patients described the term safety using words associated with cleanliness such as “sterilized or clean instruments.” Wearing clean gloves and sterilizing instruments were perceived as important hygienic practices for practitioners to follow. Additional research has concluded that patients view the cleanliness of their units and sterilization protocols, along with maintaining a “clean clinical environment,” as crucial components of patient safety and quality of care [20–22]. Other studies have reported that patients deemed the use of state-of-the-art equipment as a necessary requirement for ensuring patient safety [20].

Patients’ expectations for quality of care from their practitioners varied depending on the dental setting they attended. Our previous work found that patients belonging to academic care settings were concerned about how the inexperience of student providers might impact the quality of dental care they received; however, such apprehensions were eased by faculty member oversight [8]. This finding aligns with results from our present study where patients expressed comfort in receiving care at the academic dental care setting and were more forgiving of mistakes. Another previous study [21] revealed that the perceived clinical ability of a dental student and the presence of supervisor oversight and assistance with procedures played a critical role in reducing patient anxiety. However, these opinions were not shared by patients at private practices and the community health center in this study, because they expected higher standards of care from their providers and were less forgiving of mistakes.

Our study revealed that patients from the private dental clinic focused on the credentials and training of their providers and only felt safe if the provider had years of experience and extensive training from top tier institutions. This finding is reinforced by prior research that concludes most patients prefer older practitioners whom they perceive as more experienced with refined communication skills [20]. On the contrary, a separate study found that some patients preferred younger dentists due to their utilization of technology and innovative methods during treatments [22]. This study also found that patients preferred female dentists because they believed that they had better interpersonal skills than their male counterparts. Together, these studies highlight how the perceived skills of dentists and their demographic characteristics can impact a patient’s perception of receiving quality dental services [23].

Dental practitioners have experienced a high volume of complaints over the years. Different studies have found that these complaints originate from various sources. While one study found most complaints were made by parents or relatives of patients [24], another reported that the majority of complaints received were about personal dental treatment [25]. Nonetheless, most complaints were made by women [25]. Reasons for complaints included: post-treatment symptoms like pain and eating issues, emotional trauma, unprofessional conduct, and communication breaches [25]. In the present study, patients expressed the importance of having a patient advocate who could help them discuss negative experiences with their dentists. Others preferred to seek an alternative dentist rather than return to the same practitioner following an adverse event. Fear played a major role as patients decided whether to report their experiences at the dentist. These findings are in accordance with other studies where patients reported losing trust in their dentist and changing providers due to adverse incidents or perceived risks. However, most patients were able to report incidents through advocate mediums that helped advocate for financial compensations and detect preventable injuries [25].

Our study also revealed that clear and concise communication was an important strategy for improving the quality of patient care. Participants recommended that practitioners engage in open conversations with patients and give honest opinions on their current oral health status and treatment recommendations. They noted that these strategies could encourage active patient participation in promoting quality and safety by enabling them to make informed decisions. Given the importance of conveying information pre-, during, and post-treatment, study participants viewed failure to properly communicate as a major cause for concern. Similar findings have been reported in other studies, Caltabiano et al [21].found that 50% of dental patients cited “interpersonal skills” of dental students as a factor that decreased anxiety among their dental patients. Simple descriptions of a patient’s diagnosis and the available treatment options are necessary to attain patient satisfaction and participation [26]. Research studies have shown that clear explanations during consultations and active listening to patients enable them to grasp the expected outcome of the proposed treatment. Miscommunication, rudeness, and inattentiveness can cause a breach in the relationship between dentists and patients [27]. Adequate communication is necessary to properly assess a patient’s medical condition or medication use before treatment and to help manage patient behavior during treatments to prevent adverse incidents [28]. Unclear explanations or indications by professionals can result in poor treatment adherence by patients, thereby compromising effectiveness [20]. Findings from other studies revealed that patients considered good dental services to include key communication strategies such as empathetic words of encouragement and comfort during the treatment process [23]. A prior study showed that 40% of patients undergoing dental radiographic treatments never had their dentist explain negative side effects and risk of treatment. More than half of these patients (55%) never or hardly ever made enquiries into the safety measures before undergoing radiography [29].

Involving patients in the monitoring and reporting process gives them a key role to play in enhancing patient safety, as they can provide provider feedback and report adverse incidents [28]. Patients in the current study expressed willingness to participate in focus groups that allow them to voice their opinions without fear of repercussions from dentists. Similarly, another study found that patients were willing to actively participate in their care and safety by advocating for themselves and being involved in the decision-making process regarding their conditions. Patients’ participation in care and patient safety measures were used as determinants to assess whether they felt safe or ignored [30].

Different patients preferred different forms of communication based on certain demographic factors. Older patients preferred to receive printed copies of ‘before’ and ‘after-visit’ summaries and were not comfortable using technological gadgets, while younger patients preferred iPads and other mobile devices, as they considered them to be more effective educational devices for patient engagement during treatment. Though most participants were female, there was no gender-based differences of opinions. A study of internal medicine patients found no difference in participation in patient safety activities based on age, gender, or profession [18]. However, an alternative study indicated that younger patients with advanced education were more willing to participate in the decision-making process regarding their treatments [20].

Although our findings have limited generalizability due to the use of convenience sampling, our study provides critical information on the willingness of dental patients across various dental care settings to participate in activities that promote the quality and safety of dental care. This study builds upon the findings from our previous work investigating patient participation at a single academic dental center. The conclusions confirm that dental patients react differently to working as partners depending on the dental care setting in which they receive care. Future studies assessing the patient’s perspective should also assess their oral health literacy, since their knowledge of dentistry may impact their perception. Such studies will help determine accessible and feasible methods for improving patient engagement in quality and safety.

Based on our findings, we offer several recommendations on how to facilitate patient participation in safety and quality care activities. “What to expect” summaries for pre-, during, and post-dental treatment periods need to be developed and customized for each dental procedure. Practices should consider employing patient advocates to handle patient concerns and make the feedback process more approachable. While employing a patient advocate might not be feasible in smaller dental offices, some alternatives could be to designate an administrative staff member to manage patient concerns or partner with other local dental offices to outsource the handling of patient grievances to a third-party patient advocacy or mediation group for resolution. Results from our study emphasize that dental practitioners should be approachable and deliver the necessary information at the right time using various modalities depending on the patient’s needs and preferences. Altogether, implementing these strategies will improve patient participation in quality and safety activities in dental care settings.

## Conclusion

Our study revealed that dental patients care about the quality and safety of care that they receive. Their willingness to participate in quality and safety activities depended on their relationship with the provider and their perception regarding the receptiveness of providers to accept feedback. Patients were less willing to participate if an activity if it could potentially be perceived as confrontational. The type of dental care setting slightly impacted how patients perceived their role as partners in improving the quality and safety of dental care.

### Supplementary Information

**Supplementary Material 1.**

**Supplementary Material 2.**

## Data Availability

The datasets during and/or analyzed during the current study available from the corresponding author on reasonable request.

## References

[1] Bailey E, Tickle M, Campbell S, O’Malley L (2015). Systematic review of patient safety interventions in dentistry. BMC Oral Health.

[2] Black I, Bowie P (2017). Patient safety in dentistry: development of a candidate 'never event' list for primary care. Br Dent J.

[3] Kalenderian E, Obadan-Udoh E, Maramaldi P (2017). Classifying adverse events in the dental office.

[4] Kalenderian E, Obadan-Udoh E, Yansane A (2018). Feasibility of electronic health record–based triggers in detecting dental adverse events. Appl Clin Inform..

[5] Maramaldi P, Walji MF, White J (2016). How dental team members describe adverse events. J Am Dent Assoc.

[6] Obadan EM, Ramoni RB, Kalenderian E (2015). Lessons learned from dental patient safety case reports. J Am Dent Assoc.

[7] Obadan-Udoh E, Panwar S, Yansane A-I (2020). Are dental patients concerned about safety? An exploratory study. J Evid Based Dent Pract.

[8] Obadan-Udoh EM, Gharpure A, Lee JH, Pang J, Nayudu A (2021). Perspectives of dental patients about safety incident reporting: a qualitative pilot study. J Patient Saf.

[9] Wright S, Ucer C, Speechley S (2017). The perceived frequency and impact of adverse events in dentistry. Fac Dent J.

[10] Walji M, Yansane A, Hebballi N (2020). Finding dental harm to patients through electronic health record-based triggers. JDR Clin Transl Res.

[11] Kalenderian E, Obadan-Udoh E, Maramaldi P (2021). Classifying adverse events in the dental office. J Patient Saf.

[12] Franklin A, Kalenderian E, Hebballi N (2022). Building consensus for a shared definition of adverse events: a case study in the profession of dentistry. J Patient Saf.

[13] Kalenderian E, Lee JH, Obadan-Udoh EM, Yansane A, White JM, Walji MF (2022). Development of an inventory of dental harms: methods and rationale. J Patient Saf.

[14] Yansane A, Lee J, Hebballi N (2020). Assessing the patient safety culture in dentistry. JDR Clin Transl Res.

[15] Vincent CA, Coulter A (2002). Patient safety: what about the patient?. Qual Saf Health Care.

[16] Davis RE, Sevdalis N, Pinto A, Darzi A, Vincent CA (2013). Patients’ attitudes towards patient involvement in safety interventions: results of two exploratory studies. Health Expect.

[17] Davis RE, Sevdalis N, Vincent CA (2011). Patient involvement in patient safety: How willing are patients to participate?. BMJ Qual Saf..

[18] Sahlström M, Partanen P, Azimirad M, Selander T, Turunen H (2019). Patient participation in patient safety—an exploration of promoting factors. J Nurs Manag..

[19] Weingart SN, Price J, Duncombe D (2007). Patient-reported safety and quality of care in outpatient oncology. Jt Comm J Qual Patient Saf.

[20] Henríquez-Tejo RB, Cartes-Velásquez RA (2016). Patients’ perceptions about dentists: a literature review. Odontoestomatologia.

[21] Caltabiano ML, Croker F, Page L (2018). Dental anxiety in patients attending a student dental clinic. BMC Oral Health.

[22] Furnham A, Swami V. Patient preferences for dentists. Psychol Health Med. 2009;14(2):143–9. 10.1080/13548500802282690 19235073

[23] Luo JYN, Liu PP, Wong MCM (2018). Patients’ satisfaction with dental care: a qualitative study to develop a satisfaction instrument. BMC Oral Health.

[24] Thomas L, Tibble H, Too L, Hopcraft M, Bismark M (2018). Complaints about dental practitioners: an analysis of 6 years of complaints about dentists, dental prosthetists, oral health therapists, dental therapists and dental hygienists in Australia. Aust Dent J.

[25] Hiivala N, Mussalo-Rauhamaa H, Murtomaa H (2015). Can patients detect hazardous dental practice? A patient complaint study. Int J Health Care Qual Assur..

[26] Iqbal W, Faran F, Yashfika A, Shoro F (2018). Evaluation of dental care through patient satisfaction feedback–a cross sectional study at Dental Institute of OJHA Hospital, Karachi. Pakistan Adv Dent & Oral Health.

[27] Dental Protection Limited. Handling Compliants-England. https://www.dentalprotection.org/docs/librariesprovider4/dental-advice-booklets/dental-advice-booklet-complaints-handling-england.pdf. Published 2016. Accessed 25 July 2022.

[28] Hiivala N (2016). Patient safety incidents, their contributing and mitigating factors in dentistry.

[29] Al Faleh W, Mubayrik AB, Al Dosary S, Almthen H, Almatrafi R (2020). Public perception and viewpoints of dental radiograph prescriptions and dentists’ safety protection practice. Clin Cosmet Investig Dent.

[30] Ringdal M, Chaboyer W, Ulin K, Bucknall T, Oxelmark L (2017). Patient preferences for participation in patient care and safety activities in hospitals. BMC Nurs.

