# Supplementary material for: Dental patients as partners in promoting quality and safety: a qualitative exploratory study
Source: BMC Oral Health. 2024 Apr 10;24:438. doi: 10.1186/s12903-024-04030-1 (PMC11005277; doi:10.1186/s12903-024-04030-1)
Supplement: Supplementary file 1 — Supplementary Material 1. [file 12903_2024_4030_MOESM1_ESM.docx]

**Focus Group Discussion Guide**

**Welcome, Consent Process and Introductions**

1. **Welcome**

Welcome and thank you for volunteering to take part in this focus group. We understand that you are busy and we appreciate your time. Let me introduce myself. My name is Dr. Enihomo Obadan-Udoh and I will be the moderator in today’s discussion. It is my job to make sure that everyone here gets to participate and that we stay on track. I will be assisted by ­­­­_______ who will record and summarize your responses.

In this focus group we will discuss patient engagement in quality and safety activities at the dental office. We believe that as a dental patient, your perspective is vital to ensuring that we deliver the highest quality of care and maintain a safe environment at the dental office. Specifically, we would like to know your thoughts and ideas about how willing patients are to participate in activities that promote quality and safety, the extent of that willingness, and the strategies that will enhance or reduce this willingness to participate. Our ultimate goal is to achieve better patient experiences and better health outcomes.

1. **Consent Process**

All of you should have received a copy of the consent form and had some time to review it. I would like to go over the document one more time to be sure that you understand all aspects of the study and what your rights are as a participant. [Confirm that everyone has signed in and completed the demographic & pre-session questionnaire]

If there are any questions that you do not wish to answer, you do not have to do so; however, we encourage you to be as open and truthful as possible.

1. **Warm up**

First, I’d like everyone to introduce themselves. Can you tell us your first name?

**Focus group Logistics and Ground rules:**

1. **Logistics**

- This focus group session will last about one and a half hours.
- You are free to use the bathroom or help yourself to extra food/drinks during the session.

1. **Ground Rules**

By show of hands, has anyone participated in a focus group before? There are certain ground rules that we must follow

- The most important rule is that only one person speaks at a time. There may be a temptation to jump in when someone is talking but please wait until they have finished.
- We want everyone to participate; every person's experiences and opinions are valuable.
- There are no right or wrong answers – We want you to speak up whether you agree or disagree. We want to hear a wide range of opinions.
- You do not have to speak in any particular order.
- You do not have to agree with the views of other people in the group; We are not trying to achieve consensus, we’re gathering information
- In addition to the handwritten notes that will be taken by the investigator and assistant, this session will also be audio recorded to get a better understanding of your responses and to double check our data for accuracy.
- Please refrain from any side conversations and speak clearly so we can record your responses properly.
- Please refrain from discussing any personal details shared here with persons outside of this focus

group.

- Does anyone have any questions? [Answers].

• Before we get started, I want you to assure you that all comments made during this session will be kept completely confidential. We will not identify anyone by name in our report. All audio recordings will be stored securely on encrypted devices and password-protected laptops.

**May I start the tape recorder? (If yes, switch on the recorder)**

- OK, let’s begin

*[Discussion begins, make sure to give people time to think before answering the questions and don’t move too quickly. Use the probes to make sure that all issues are addressed, but move on when you feel you are starting to hear repetitive information].*

**Focus group questions:**

1. **What do you understand by the terms ‘patient safety’ and ‘quality’ – what do you think I mean/I am talking about when I use these terms?**

*For the purpose of our discussion, we will define…*

*Safety: Freedom from accidental injury or harm*

*Quality: The degree to which health care services increase the likelihood of desired health outcomes and are consistent with current professional knowledge-safe, efficient, effective, equitable, timely, and patient-centered.*

1. **What kinds of things do dental offices and dentists, hygienists, and dental assistants currently do to keep patients safe or to ensure high quality care?**
   1. Have you experienced any initiative or campaign at the dental office?
   2. What about at the medical office with your physicians e.g. How many of you have heard about the PINK video, or “Speak Up” or “Five Moments of Hand Hygiene”
   3. Should dental offices be doing more than they are currently doing?
2. **How do you feel about the idea that patients can contribute to their safety or the quality of care they receive?**
   1. What sorts of things can patients [carers, relatives] do to help keep themselves safe?
   2. How confident or comfortable would patients feel about doing any of these things?
   3. What sorts of things do you think might stop patients/put them off/encourage them to do any of the things you suggest?
   4. Are there any benefits to participating despite these barriers?
   5. How do you think the dental staff might feel about the efforts you describe/suggest?
3. **What approaches can be used to improve the engagement of dental patients in quality and safety activities at the dental office?**
   1. Education campaigns to raise awareness and inform patients of tangible ways to get involved. Posters, flyers, brochures
   2. Engaging patients and family members upfront through focus groups,
   3. Involvement in the Quality committees, special taskforces, to identify quality and safety issues
   4. Involving patient in research design about quality and safety
   5. Shared-decision making between dentists and patients.i.e. dedicated time during appointments to discuss the quality and safety of care?
   6. Other suggestions?

**How would you rank these activities in terms of their effectiveness?**

1. **What factors would affect your willingness to participate in any of these quality and safety activities at the dental office?**
   1. Are there particular activities that you would feel more comfortable or less comfortable participating in?
   2. Does the type of question affect your willingness to participate? For example, the questionnaire was divided into factual vs challenging questions; interactional vs non-interactional questions. Which of these were you more likely to do and why?
   3. Does the type of healthcare professional who invites you to participate make a difference? Why or why not?
   4. Does the nature of your illness or complexity of your procedure affect whether or not you would be willing to engage in these activities?
2. **In what situations would you be comfortable notifying a dentist or dental assistant about an experience that you perceive to be poor quality or unsafe?** [ask for specific examples]

Quality: technical/procedural error, service quality?

Safety: Harm-Pain? Bleeding? Infection, nerve damage, WSPP, Systemic effects etc?

1. What would affect your decision to notify the provider about this experience?
2. Are there any benefits to patients for reporting safety-related incidents to a national database?

[For example: to Improve health outcomes? Prevent future mistakes?]

Probe: Are there any benefits to others? E.g. clinic? Providers? Other patients?

1. **How would you describe a “safe space” to report any incident that affects the quality or safety of care that you receive? what are your expectations from such a system?**

Probe:

- 1. What will the features be?
  2. Which medium would be best for you? e.g. Waiting room Kiosk? Anonymous form? After-visit surveys? Mobile app? Website? Positive reviews only? Negative reviews only?
  3. What type of feedback do you expect to receive and in what time frame?
  4. What would you hope this information would be used for?

1. **How do you currently assess the quality or safety of dental offices in general/before you choose one?**
   1. What information would you wish were available when assessing the quality or safety of care before choosing a dental office/provider?
   2. What information would positively or negatively affect your assessment of the quality or safety rating of any dental office/decision to visit a dental office?
2. **Anything else you would like to add to our discussion today?**

That concludes our focus group. Thank you so much for coming and sharing your thoughts and opinions with us. If you have additional information that you did not get to say in the focus group, please feel free to let us know.
