# Supplementary material for: Dental patients as partners in promoting quality and safety: a qualitative exploratory study
Source: BMC Oral Health. 2024 Apr 10;24:438. doi: 10.1186/s12903-024-04030-1 (PMC11005277; doi:10.1186/s12903-024-04030-1)
Supplement: Supplementary file 2 — Supplementary Material 2. [file 12903_2024_4030_MOESM2_ESM.docx]

**Informational Survey**

*Please select the option (from 1= “Definitely Will” through 7= “Definitely Not”) that best describes your willingness to engage in the following activities at this dental office.*

| **Section 1a: Factual Questions to a Dental Assistant/Hygienist (Interactional Behavior)** | **Definitely**  **Will** | **Probably**  **Will** | **Possibly Will** | **Neutral** | **Possibly Not** | **Probably Not** | **Definitely Not** |  |
| --- | --- | --- | --- | --- | --- | --- | --- | --- |
| 1. Would you ask a dental assistant/hygienist: How long will this dental procedure last? | 1 | 2 | 3 | 4 | 5 | 6 | 7 |  |
| 1. Would you ask a dental assistant/hygienist: How long will the pain last after the procedure? | 1 | 2 | 3 | 4 | 5 | 6 | 7 |  |
| 1. Would you ask a dental assistant/hygienist: What signs should I look out for if my teeth/gums are not healing as they should? | 1 | 2 | 3 | 4 | 5 | 6 | 7 |  |
| 1. Would you ask a dental assistant/hygienist: When can I resume eating and drinking? | 1 | 2 | 3 | 4 | 5 | 6 | 7 |  |
| 1. Would you ask a dental assistant/hygienist: How is the procedure (e.g. scaling and polishing) performed? | 1 | 2 | 3 | 4 | 5 | 6 | 7 |  |
| 1. If a dental assistant/hygienist encouraged you to ask the above questions (e.g. by saying “it’s ok to ask staff questions”), would you be more willing to ask these questions? | 1 | 2 | 3 | 4 | 5 | 6 | 7 |  |
|  | | | | | | | |  |
| **Section 1b: Factual Questions to a Dentist (Interactional Behavior)** | **Definitely**  **Will** | **Probably**  **Will** | **Possibly Will** | **Neutral** | **Possibly Not** | **Probably Not** | **Definitely Not** |  |
| 1. Would you ask a dentist: How long will the pain last after the procedure? | 1 | 2 | 3 | 4 | 5 | 6 | 7 |  |
| 1. Would you ask a dentist: How long will this dental procedure last? | 1 | 2 | 3 | 4 | 5 | 6 | 7 |  |
| 1. Would you ask a dentist: When can I resume eating and drinking? | 1 | 2 | 3 | 4 | 5 | 6 | 7 |  |
| 1. Would you ask a dentist: How is the procedure (e.g. root canal treatment) performed? | 1 | 2 | 3 | 4 | 5 | 6 | 7 |  |
| 1. Would you ask a dentist: What signs should I look out for if my teeth/gums are not healing as they should? | 1 | 2 | 3 | 4 | 5 | 6 | 7 |  |
| 1. If a dentist encouraged you to ask the above questions (e.g. by saying “it’s ok to ask dentists’ questions”), would you be more willing to ask these questions? | 1 | 2 | 3 | 4 | 5 | 6 | 7 |  |
|  | | | | | | | | |
| **Section 2a: Challenging Questions to a Dental Assistant/Hygienist (Interactional Behavior)** | **Definitely**  **Will** | **Probably**  **Will** | **Possibly Will** | **Neutral** | **Possibly Not** | **Probably Not** | **Definitely Not** |  |
| 1. Would you ask a dental assistant/hygienist: Can you check that you have the correct tooth site/location for my procedure? | 1 | 2 | 3 | 4 | 5 | 6 | 7 |  |
| 1. Would you ask a dental assistant/hygienist: How many of these procedures have you performed? | 1 | 2 | 3 | 4 | 5 | 6 | 7 |  |
| 1. Would you ask a dental assistant/hygienist: Why are you using that instrument/piece of equipment? | 1 | 2 | 3 | 4 | 5 | 6 | 7 |  |
| 1. Would you ask a dental assistant/hygienist: Have you washed your hands? | 1 | 2 | 3 | 4 | 5 | 6 | 7 |  |
| 1. Would you ask a dental assistant/hygienist: What are the risks, benefits, and alternatives of this procedure? | 1 | 2 | 3 | 4 | 5 | 6 | 7 |  |
| 1. If a dental assistant/hygienist encouraged you to ask the above questions (e.g. by saying “it’s ok to ask staff questions”) would you be more willing to ask these questions? | 1 | 2 | 3 | 4 | 5 | 6 | 7 |  |
|  | | | | | | | | |
| **Section 2b: Challenging Questions to a Dentist (Interactional Behavior)** | **Definitely**  **Will** | **Probably**  **Will** | **Possibly Will** | **Neutral** | **Possibly Not** | **Probably Not** | **Definitely Not** |  |
| 1. Would you ask a dentist: Why are you using that instrument/piece of equipment? | 1 | 2 | 3 | 4 | 5 | 6 | 7 |  |
| 1. Would you ask a dentist: Can you check that you have the correct tooth site/location for my procedure? | 1 | 2 | 3 | 4 | 5 | 6 | 7 |  |
| 1. Would you ask a dentist: How many of these procedures have you performed? | 1 | 2 | 3 | 4 | 5 | 6 | 7 |  |
| 1. Would you ask a dentist: Have you washed your hands? | 1 | 2 | 3 | 4 | 5 | 6 | 7 |  |
| 1. Would you ask a dentist: What are the risks, benefits, and alternatives of this procedure? | 1 | 2 | 3 | 4 | 5 | 6 | 7 |  |
| 1. If a dentist encouraged you to ask the above questions (e.g. by saying “it’s ok to ask dentists’ questions”) would you be more willing to ask these questions? | 1 | 2 | 3 | 4 | 5 | 6 | 7 |  |
|  | | | | | | | | |
| **Section 3a: Notifying Dental Assistant/Hygienist (Interactional Behavior)** | **Definitely**  **Will** | **Probably**  **Will** | **Possibly Will** | **Neutral** | **Possibly Not** | **Probably Not** | **Definitely Not** |  |
| 1. Would you notify a dental assistant/hygienist if you thought your gums/mouth had become infected after a procedure? | 1 | 2 | 3 | 4 | 5 | 6 | 7 |  |
| 1. Would you notify a dental assistant/hygienist if they had the dental record/radiograph of the wrong patient pulled up while discussing your treatment plan? | 1 | 2 | 3 | 4 | 5 | 6 | 7 |  |
| 1. Would you notify a dental assistant/hygienist if you did not receive the results of a biopsy test for an oral swelling/mass? | 1 | 2 | 3 | 4 | 5 | 6 | 7 |  |
| 1. Would you notify a dental assistant/hygienist if you thought an error had occurred in your care? | 1 | 2 | 3 | 4 | 5 | 6 | 7 |  |
| 1. Would you notify your dental assistant/hygienist if there has been a significant change in your medical history? | 1 | 2 | 3 | 4 | 5 | 6 | 7 |  |
| 1. For the above problems and concerns, if a dental assistant/hygienist said to you “it’s ok to notify me of any of these problems or errors”, would you be more willing to do this? | 1 | 2 | 3 | 4 | 5 | 6 | 7 |  |
|  | | | | | | | | |
| **Section 3b: Notifying Dentist (Interactional Behavior)** | **Definitely**  **Will** | **Probably**  **Will** | **Possibly Will** | **Neutral** | **Possibly Not** | **Probably Not** | **Definitely Not** |  |
| 1. Would you notify a dentist if you did not receive the results of a biopsy test for an oral swelling/mass? | 1 | 2 | 3 | 4 | 5 | 6 | 7 |  |
| 1. Would you notify a dentist if you thought an error had occurred in your care? | 1 | 2 | 3 | 4 | 5 | 6 | 7 |  |
| 1. Would you notify a dentist if you thought your gums/mouth had become infected after a procedure? | 1 | 2 | 3 | 4 | 5 | 6 | 7 |  |
| 1. Would you notify a dentist if they had the dental record/radiograph of the wrong patient pulled up while discussing your treatment plan? | 1 | 2 | 3 | 4 | 5 | 6 | 7 |  |
| 1. Would you notify your dentist if there has been a significant change in your medical history? | 1 | 2 | 3 | 4 | 5 | 6 | 7 |  |
| 1. For the above problems and concerns, if a dentist said to you “it’s ok to notify me of any of these problems or errors”, would you be more willing to do this? | 1 | 2 | 3 | 4 | 5 | 6 | 7 |  |
|  | | | | | | | | |
| **Section 4: Information Provision (Non-Interactional Behavior)** | **Definitely**  **Will** | **Probably**  **Will** | **Possibly Will** | **Neutral** | **Possibly Not** | **Probably Not** | **Definitely Not** |  |
| 1. Would you be willing to bring into the dental office, medications that you are taking and a list of allergies? | 1 | 2 | 3 | 4 | 5 | 6 | 7 |  |
| 1. If a dentist encouraged you to bring into the dental office, medications and a list of allergies, would you be more willing to do this? | 1 | 2 | 3 | 4 | 5 | 6 | 7 |  |
| 1. If a dental assistant/hygienist encouraged you to bring into dental office, medications and a list of allergies, would you be more willing to do this? | 1 | 2 | 3 | 4 | 5 | 6 | 7 |  |
| **Section 5: Information Gain (Non-Interactional Behavior)** | **Definitely**  **Will** | **Probably**  **Will** | **Possibly Will** | **Neutral** | **Possibly Not** | **Probably Not** | **Definitely Not** |  |
| 1. Would you want to be given information to help you decide which dental office had the highest safety record for your treatment? | 1 | 2 | 3 | 4 | 5 | 6 | 7 |  |
| 1. If a dentist encouraged you to look at information to help you decide which dental office had the highest safety record, would you be more willing to do this? | 1 | 2 | 3 | 4 | 5 | 6 | 7 |  |
| 1. If a dental assistant/hygienist encouraged you to look at information to help you decide which dental office had the highest safety record, would you be more willing to do this? | 1 | 2 | 3 | 4 | 5 | 6 | 7 |  |
| **Section 6: Reporting (Non-Interactional Behavior)** | **Definitely**  **Will** | **Probably**  **Will** | **Possibly Will** | **Neutral** | **Possibly Not** | **Probably Not** | **Definitely Not** |  |
| 1. If you experienced an error in your care, would you report this to a national reporting system? | 1 | 2 | 3 | 4 | 5 | 6 | 7 |  |
| 1. If a dentist encouraged you to report an error you experienced in your care to a national reporting system, would you be more willing to do this? | 1 | 2 | 3 | 4 | 5 | 6 | 7 |  |
| 1. If a dental assistant/hygienist encouraged you to report an error you experienced in your care to a national reporting system, would you be more willing to do this? | 1 | 2 | 3 | 4 | 5 | 6 | 7 |  |

Thanks for completing this survey!
